# War in Ukraine and Racism: The Physical and Mental Health of Refugees of Color Matters

**DOI:** 10.3389/ijph.2022.1604990

**Published:** 2022-04-27

**Authors:** Jude Mary Cénat, Wina Paul Darius, Pari-Gole Noorishad, Sara-Emilie McIntee, Elisabeth Dromer, Joana Ntunga Mukunzi, Oluwafayoslami Solola, Monnica T. Williams

**Affiliations:** Interdisciplinary Centre for Black Health, Faculty of Social Sciences, University of Ottawa, Ottawa, ON, Canada

**Keywords:** Russia and Ukrain war, physical and mental health, refugees of color, racism, racial trauma

The number of refugees has reached unprecedented levels with approximately 24.6 million people fleeing persecution, including war and other forms of organized violence [1]. Refugees escaping war face horror prior, during, and following their migration journey due to violence, malnutrition, imprisonment, sexual violence, torture, loss of property and livelihood, separation or death of loved ones, and resettlement stress [2, 3]. Overwhelming research has shown that war-related trauma and refugees’ stress are associated with increased rates of both physical and mental health problems, including posttraumatic stress disorder (PTSD), depression, anxiety, psychosis, hypertension, diabetes, and cardiovascular disease [4, 5].

On February 24, 2022, Russia invaded Ukraine, leading the United Nations High Commissioner’s Refugee Council (UNHRC) to declare a Level 3 state of emergency (highest level) [6]. The conflict thus far has resulted in the destruction of infrastructure, thousands of casualties, and a high volume of displaced individuals over a record period [6]. As of April 4, 2022, about 4.2 million refugees have fled Ukraine) [6]. Moreover, 12 million Ukrainians (around 30% of the national population) are projected to need humanitarian assistance in 2022 [6]. Ukrainian refugees are primarily fleeing to Poland (2,469,657), Romania (648,410), Republic of Moldova (396,448), Hungary (394,728), Slovakia (301,405), other European countries, but also the Russian Federation (350,632) and Belarus (15,281) [6]. With this flood of refugees, difficulties crossing borders have been observed and People of Color (POC) have been particularly affected due to their skin color and ethnic origin in a way that can aggravate traumas related to the war they have experienced [7].

The war in Ukraine has shed light on multiple forms of racism—the belief that certain people are inferior because of their skin color [8]. First, journalists, political commentators, politicians, and a large part of the public have alluded that European (i.e., White) people deserve more dignity and respect than POC [9]. War in a European country has come as a surprise to many reporters, with some stating that it is not a “third-world nation.” “Other reporters expressed that “we are not in Middle East or Africa” and commented that Ukrainian refugees are “civilized,” from the “middle-class,” like “any European family,” and deserve life because they have “blue eyes and blond hair.” Through such statements, Westerners trivialize violence in other countries and against POC [10]. Second, racism also permeates at the borders between Ukraine and neighboring countries [7]. POC, especially African, Arab, and Indian international students, have reported that border guards push them to the back of lines, assault them, prevent them from boarding buses, and state that “one foreigner can leave for every hundred Ukrainian” [11]. The average time for POC to cross borders is longer compared to Ukrainians [12]. Refugees of color who have been able to cross borders find it more difficult to find temporary housing and assistance in European countries [13]. Hence, this pervasive racism opens doors for some on the basis of skin, eyes, and hair color whereas it compromises the health and lives of thousands of Black, Arab, Indian, and Pakistani refugees, among others. Third, European politicians with anti-immigration stances have also tied the right to live to skin color and culture. They have shown differential treatment toward refugees by historically blocking entry to refugees of color from varying countries but welcoming White Ukrainian refugees without hesitation [7, 14]. Refugees of color from Ukraine now carry a double trauma: the burden of war in addition to racism. War is a highly traumatic experience that has detrimental impacts on physical and mental health resulting in problems such as injuries, anxiety, depression, PTSD, hypertension, diabetes, and cardiovascular disease [5, 15]. Refugees of color experiencing racial trauma before and after crossing borders may experience exacerbated consequences. Racial trauma refers to dangerous experiences related to threats, prejudices, harm, shame, humiliation, and guilt associated with various types of racial discrimination experienced either as a victim or as a witness [8]. It has been associated with PTSD, depression, anxiety, increased suicidal ideation and attempts, hypertension, diabetes, heart disease, etc. [4, 15]. The addition of physical and mental health problems related to war, racism, and the current COVID-19 pandemic can further deteriorate the health of refugees of color. The racism experienced by refugees of color may also impact the health of POC in Western countries who were outraged by the racist images and comments of journalists, political commentators, and politicians. It may also impact their sense of belonging.

Citizenship and belonging remain debated concepts. The racism experienced by refugees of color during the ongoing Russian invasion of Ukraine further complicates POC’s conceptualizations of belonging to Western societies. The racist treatment experienced by refugees of color at different borders constitutes a crack in the Western unity against the Russian regime. Racial tensions at European borders highlighted once again the unfairness of certain Western ideologies and policies. If the West can achieve unity in the face of violence, why then is that same compassion not extended to non-White refugees? The treatment of refugees of color has impacted the remaining faith of POC in Western democracies [16]. The more these groups are ostracized and othered, the less merit they will find in the values of a democracy [16]. This has impacted their sense of belonging, provoked neutral opinion about this war among some POC communities, and some now support the Russian invasion of Ukraine.

Some may think that discussions on the inhumane treatment of refugees of color can wait until after the war in Ukraine has ended. However, human lives are at risk every second and no refugee should be denied basic rights because of interactions between their identities and others’ ideological beliefs. In addition, experiences of racism within Ukrainian borders not only impacts the health of thousands of refugees of color, but also POC in Western countries. Solidarity movements are occurring in Canada, the United Kingdom, France, and the United States among POC communities in direct support of refugees of color [13]. We should all be reminded that violence and racism have no borders and are matters that concern us all. We urge countries that border Ukraine and those that are receiving refugees, United Nations’ agencies, non-governmental organisations, and others to provide support and care with an antiracist health approach. In addition to reducing the impact of the war on both the physical and mental health of refugees of color, an antiracist health approach toward refugees will improve POC’s views of Western democracy and show communities of color that they matter [17]. We must ensure that every person, regardless of race and culture, finds safe refuge and is welcomed with open arms. To refugees of color, we see you, we hear you, and we believe in you.

## References

[1] United Nations High Commissioner for Refugees. UNHCR - Figures at a Glance [Internet]. [cited March 13, 2022] (2022). Available from: https://www.unhcr.org/figures-at-a-glance.html .

[2] Sigvardsdotter E Vaez M Rydholm Hedman AM Saboonchi F. Prevalence of Torture and Other Warrelated Traumatic Events in Forced Migrants: A Systematic Review. Torture (2016) 26(2):41–73. 27858780

[3] Cénat JM Charles CH Kebedom P. Multiple Traumas, Health Problems and Resilience Among Haitian Asylum Seekers in Canada’s 2017 Migration Crisis: Psychopathology of Crossing. J Loss Trauma (2020) 25(5):416–37. 10.1080/15325024.2019.1703610

[4] Blackmore R Boyle JA Fazel M Ranasinha S Gray KM Fitzgerald G The Prevalence of Mental Illness in Refugees and Asylum Seekers: A Systematic Review and Meta-Analysis. PLoS Med (2020) 17(9):e1003337. 10.1371/journal.pmed.1003337 32956381 PMC7505461

[5] Carlsson J Sonne C. Mental Health, Pre-migratory Trauma and Post-migratory Stressors Among Adult Refugees. Res Clin Pract (2018) 2018:15–35. 10.1007/978-3-319-97046-2_2

[6] United Nations High Commissioner for Refugees. Situation Ukraine Refugee Situation [Internet]. [April 4, 2022] (2022). Available from: https://data2.unhcr.org/en/situations/ukraine.

[7] Howard PSS Johnson BCY Ah-Sen K. Ukraine Refugee Crisis Exposes Racism and Contradictions in the Definition of sBT. The Conversation. [cited April 11, 2022] (2022). Available from: https://theconversation.com/ukraine-refugee-crisis-exposes-racism-and-contradictions-in-the-definition-of-human-179150.

[8] Haeny AM Holmes SC Williams MT. The Need for Shared Nomenclature on Racism and Related Terminology in Psychology. Perspect Psychol Sci (2021) 16(5):886–92. 10.1177/17456916211000760 34498528 PMC8439544

[9] Hellyer HA. Opinion Ukraine News Coverage Exposes Racist Biases in Western media - the Washington Post. Washington Post [Internet]. [cited April 11, 2022] (2022). Available from: https://www.washingtonpost.com/opinions/2022/02/28/ukraine-coverage-media-racist-biases/.

[10] Samuelson K. The Ukraine War’s Race Problem. [cited March 13, 2022]. The Week UK [Internet] (2022).

[11] Adams C Essamuah Z Walters S Abdelkader R. Open the door or we die”: Africans report racism and hostility trying to flee Ukraine. NBC News [Internet] (2022). [cited March 13, 2022]. Available from: https://www.nbcnews.com/news/nbcblk/open-door-die-africans-report-racism-hostility-trying-flee-ukraine-rcna17953.

[12] Redd K. As Long as You Are Black, No One Likes You”. Africans Fleeing Ukraine Report Racism and Discrimination at the Border | abc10.Com. [cited April 11, 2022] (2022). Available from: https://www.abc10.com/article/news/community/race-and-culture/african-students-fleeing-ukraine-say-they-are-facing-discrimination-at-the-border/103-ecd263d6-fe59-45d0-ad4b-e8199204c879 .

[13] Deachman B. Ottawa-led Coalition Helps rescue 41 Black International Students from Ukraine. Ottawa Citizen. [March 27, 2022] (2022). Available from: https://ottawacitizen-com.cdn.ampproject.org/c/s/ottawacitizen.com/news/local-news/ottawa-led-coalition-helps-rescue-41-black-international-students-from- ukraine/wcm/ddd0d260-e13b-44d4-9191-7be7f3fdf50b/amp/ .

[14] Augustová K. Why Is Europe Suddenly So Interested in Helping Refugees? Refugees. Al Jazeera [Internet]. AlJazeera. [April 11, 2022] (2022). Available from: https://www.aljazeera.com/opinions/2022/3/25/why-are-europeans-suddenly-so-interested-in-helping-refugees .

[15] Lebano A Hamed S Bradby H Gil-Salmerón A Durá-Ferrandis E Garcés-Ferrer J Migrants' and Refugees' Health Status and Healthcare in Europe: a Scoping Literature Review. BMC Public Health (2020) 20(1):1039. 10.1186/s12889-020-08749-8 32605605 PMC7329528

[16] Quarcoo A. Global Democracy Supporters Must Confront Systemic Racism. Carnegie Endow Int Peace (2020).

[17] Cénat JM. How to Provide Anti-racist Mental Health Care. The Lancet Psychiatry (2020) 7(11):929–31. 10.1016/S2215-0366(20)30309-6 32652050

